# Mass Transfer Study on Improved Chemistry for Electrodeposition of Copper Indium Gallium Selenide (CIGS) Compound for Photovoltaics Applications

**DOI:** 10.3390/nano11051222

**Published:** 2021-05-06

**Authors:** Mahfouz Saeed, Omar Israel González Peña

**Affiliations:** 1 Department of Environmental Engineering, A’Sharqiyah University, P.O. Box 42, Ibra 400, Oman; 2 Department of Chemical & Biomolecular Engineering, Case Western Reserve University, Cleveland, OH 44106, USA; 3 Water Center for Latin America and the Caribbean, School of Engineering and Sciences, Tecnologico de Monterrey, Eugenio Garza Sada Sur Avenue 2501, Colonia Tecnológico, Monterrey 64849, NL, Mexico

**Keywords:** rotating disk electrode, photovoltaic, electrodeposition, mass transport, thin film, hydrogen evolution, CIGS, PV cells, fill factor, quantum efficiency

## Abstract

Copper indium gallium selenium (CIGS) films are attractive for photovoltaic applications due to their high optical absorption coefficient. The generation of CIGS films by electrodeposition is particularly appealing due to the relatively low capital cost and high throughput. Numerous publications address the electrodeposition of CIGS; however, very few recognize the critical significance of transport in affecting the composition and properties of the compound. This study introduces a new electrolyte composition, which is far more dilute than systems that had been previously described, which yields much improved CIGS films. The electrodeposition experiments were carried out on a rotating disk electrode, which provides quantitative control of the transport rates. Experiments with the conventional electrolyte, ten times more concentrated than the new electrolyte proposed here, yielded powdery and non-adherent deposit. By contrast, the new, low concentration electrolyte produced in the preferred potential interval of −0.64 ≤ E ≤ −0.76 V vs. NHE, a smooth and adherent uniform deposit with the desired composition across a broad range of rotation speeds. The effects of mass transport on the deposit are discussed. Sample polarization curves at different electrode rotation rates, obtained in deposition experiments from the high and the low concentration electrolytes, are critically compared. Characterization of the overall efficiency, quantum efficiency, open circuit voltage, short circuit current, dark current, band gap, and the fill factor are reported.

## 1. Introduction

CuIn_x_Ga_(1−x)_Se_2_ (CIGS) is a solid thin film quaternary compound consisting of copper, indium, gallium, and selenium. In addition, CIGS is a highly effective absorber layer for photovoltaic (PV) devices. The value designated by “*x*” in the formula can range from zero to one and affects mostly the CIGS structure and its band gap energy [1]. It has been reported that the optimal *x* value is 0.3 [1]. CIGS thin film should be in the order of 1.5 to 2.5 μm; moreover, this film is attractive for photovoltaic applications because of its high optical absorption coefficient [1], about 10^5^ cm^−1^. The PV cells with CIGS as an absorption layer have an efficiency approaching 20% as has been demonstrated in laboratory tests [2]. Vacuum deposition techniques provided the best method to obtain the best CIGS absorber layer and the most efficient CIGS devices between other fabrication methods [3,4,5]. Germany’s Center for Solar Energy claims to have the record efficiency of 22.6% as it is reported in 2016 [6].

Non-vacuum techniques for fabricating CIGS films are gaining interest since they offer significantly less costly production and easier scalability to fabricate them in large areas [6,7,8,9,10,11,12,13,14,15,16,17,18,19,20,21,22,23,24,25]. Among those, the generation of CIGS thin layers by electrodeposition is particularly attractive due to the relatively low capital cost and high throughput [8,9]. The hybrid process, combining electrodeposition with metal addition from the gas phase, provided a 15.4% efficient device [10]. The introduction of Hydrion buffer, which is a mixture of sulfamic acid and potassium biphthalate in the electrolyte bath, has been particularly helpful in reducing indium and gallium oxides and hydroxides species, leading to a more stable absorber layer [10,11]. Different substrates have been reported as adequate back contacts, including molybdenum, copper, nickel, and stainless steel due to their conductivities [12,13]. Likewise, there was a study of the roughness of the electrodeposition of CIGS on different back contacts, such as fluorine tin oxide, fluorine-doped thin oxide, and molybdenum [14]. Moreover, the electrodeposition of CIGS was also studied over a ZnO window layer [15]. The electrodeposition of CIG (copper, indium, and gallium) followed by selenium addition through evaporation has also been reported [16,17]. Even an electrodeposition study of CIGS on patterned molybdenum/glass substrates showed a semi-transparent glazing-based PV cell [18]. Several research groups have focused on characterizing the chemistry of the electrodeposition bath and the growth mechanisms of the CIGS absorber [19,20,21,22,23,24]. The film produced by low temperature through an electrodeposition method exhibits low crystallinity and requires a post-electrodeposition thermal treatment (annealing) [25]. Annealing under a selenium atmosphere has been recommended in literature to accommodate the reactions of selenium with copper, gallium, and indium to generate the formation reactions, a good recrystallization, and the adjustment of the absorber’s final composition [26,27,28,29,30]. In addition, studies of the annealing process on CIGS note that the annealing step takes an important role in the efficiency of the PV cell due to it affecting the grain size and crystallinity on the absorber alloy in collecting more light [31]; in addition, the Cu/In ratio and the type of precursor can affect the morphology of the film [32]. Pulse electrodeposition was suggested to obtain a smoother CIGS layer and it improves the control in the electrodeposition [33,34,35]. An electrodeposition process followed by physical vapor deposition with an addition of the vapor phase of copper, indium, and gallium to the absorber layer has also been reported [36]. Exhaustive studies of CIGS electrodeposition processes have been provided in literature without considering the hydrogen evolution [36,37,38].

The CIGS system has been studied mostly using potential sweep methods to characterize the electrochemical reduction reactions of the four components [19,39,40,41]. Recently, the electrodeposition of CIGS on a rotating disk electrode (RDE) has been studied by two methods: DC electrodeposition and DC electrodeposition plus mechanical perturbations [42]. Furthermore, some studies have been done on a RDE on the copper-indium-selenium (CIS) system [43,44]. This means that most reported CIGS electrodeposition studies were carried out in a beaker under ill-defined transport conditions. Consequently, there is a lack of information on the significance of mass transport and agitation in the CIGS electrodeposition. In other words, despite the electrodeposition of CIGS having been studied for roughly 15 years, very few investigators recognize the critical significance of mass transport and agitation in the CIGS electrodeposition process as a very relevant parameter to obtain an absorption layer with a controlled process to reach a homogeneous atomic composition of the desired alloy in large scale areas. Likewise, it is important to note that in the formation of the CIGS alloy, it is necessary to apply very large overpotentials where there is a significant contribution of the reduction of hydrogen gas that determines the influence of the final composition and the morphology of the film alloy. However, despite the enormous problem of the co-evolution of hydrogen gas, for the moment, no studies have been reported to understand the phenomena associated with mass transfer and/or the kinetics of the reaction that governs each of these precursors separately to form the CIGS alloy. As a result, these enormous issues can be described as a big white elephant in the room that has not been addressed by previous studies. This means that the considerable amount of hydrogen gas produced in the electrodeposition of CIGS and the mass transport effect in the reduction of the alloy are elements that this study is focused on addressing. Therefore, this work provides a systematic study on the application of the RDE for characterizing the significance of transport in CIGS electrodeposition. The RDE provides a quantitative measure of the transport rates as a function of the disk rotating speed, offering well-quantified and uniform transport rates across the entire electrode.

The bath concentration in this study was reduced by a factor of ten as compared to conventional systems, providing much improved deposit properties. An investigation of the ionic mass transport in both the conventional bath and the new dilute system, with the objective of improving the film physical and optical properties, is introduced herein. Moreover, an analysis of the role taken by the hydrogen co-evolution in the electrodeposition of CIGS is done by evaluating the hydrogen current density with respect to the current density of the precursors and the total current density of the alloy reduction.

In addition to the CIGS absorber layer, an entire photovoltaic device, consisting of the layers stainless steel/Mo/Ni/CIGS/CdS/ZnO/ZnO-Al, has been fabricated using only electrodeposition methods. Consequently, quantum efficiency measurements and data collected from a solar simulator were obtained to characterize the integrated photovoltaic device. Therefore, the characterized parameters are overall efficiency, quantum efficiency, open circuit voltage, short circuit current, black current, band gap, and the fill factor.

## 2. Materials and Methods

### 2.1. Fabrication Process

Laboratory experiments were conducted using a RDE system. The system consisted of a 0.32 cm^2^ stainless-steel (406 SS). This stainless steel electrode was deposited with a layer of molybdenum via electron-beam physical vapor deposition, using 99.95% purity Mo pellets; after that a nickel deposit was also added via electrodeposition process by a rotation rate of 400 rpm at room temperature. The electrolyte composition was: 0.15 M NiSO_4_, 0.2M Na_2_SO_4_, and the pH of the bath was adjusted by sulfuric acid to 2.2; the suitable nickel electrodeposition potential applied was −1.35 V vs. SCE for 25 min to obtain a smooth layer. The nickel layer thickness estimated using a profilometer was between 1.5 to 2 µm.

The process was carried out under a vacuum atmosphere (2 × 10^−6^ torr). The deposition rate was low (0.3 A/s) to get rid of high temperature effects on the equipment. As a result, the sputtering process was carried out for 60 min to obtain a thickness of about 1 μm. After that, the disk was embedded flush in an insulating Teflon cylinder, the counter electrode was a platinum mesh, and the reference electrode was a saturated calomel electrode (SCE). These electrodes were immersed in a beaker with 50 mL within the electrochemical baths studied. The electrolyte pH was adjusted to 1.9 by the addition of hydrochloric acid. Experiments at the ambient temperature of 20 °C were carried out at rotation speeds ranging from 0 to 700 rpm.

The substrate was first rinsed with acetone, then rinsed with deionized water, and finally dried in air. Prior to electrodeposition, the substrate was electro-activated for two seconds at 1.5 V vs. NHE in 0.1 M sulfuric acid solution. The conventional higher concentration chemical system consisted of 4.2–6.5 mM CuCl_2_·2H_2_O (Sigma-Aldrich, St. Louis, MO, USA); 2.9–5 mM InCl_3_ (Strem-Chemicals, Newburyport, MA, USA); 7–9 mM H_2_SeO_3_ (Sigma-Aldrich, St. Louis, MO, USA); 3–7 mM GaCl_3_ (Strem-Chemicals, Newburyport, MA, USA); pHydrion (pH = 2) (Sigma-Aldrich, St. Louis, MO, USA); and 0.66 M LiCl (Sigma-Aldrich, St. Louis, MO, USA) as supporting electrolyte. The novel bath is at lower concentration system of 0.4–0.6 mM CuCl_2_·2H_2_O; 0.28–0.5 mM InCl_3_; 0.6–0.85 mM H_2_SeO_3_; 0.35–0.6 mM GaCl_3_; pHydrion (pH = 2); and 0.66 M LiCl as supporting electrolyte. The above-listed electrolyte ranges were explored to obtain optimal film composition and performance. The experiments were run potentiostatically, with the electrodeposition potential ranging between −0.64 V to −0.76 V vs. normal hydrogen electrode (NHE). Thus, its equivalence on the scale in the reference electrode was (0 V vs. NHE = 0.242 V vs. SCE).

In experiments where the entire PV device was fabricated, the device was completed by electrodepositing additional layers on the top of the CIGS film. The first layer on top of the CIGS absorber was about 50 nm of CdS. The cadmium sulfide electrodeposition was carried out at 500 rpm by applying −0.8V vs. SCE for 12 min, at a temperature of 65 °C from an electrolyte consisting of 0.2 M CdCl_2_, 5 mM Na_2_S_2_O_3_, and 0.5 M KCl; the bath was adjusted by HCl to 2. The CdS layer deposition was followed by 200 nm of electrodeposited un-doped zinc oxide layer. This layer was electrodeposited at 300 rpm and 75 °C by applying −0.85 V vs. SCE for 40 min from an electrolyte consisting of 0.1 M Zn(NO_3_)_2_ and 0.4 M KCl; the bath was adjusted by NaOH to 6. Lastly, a 500 nm indium-doped zinc oxide window layer was electrodeposited by applying −1.1 V vs. SCE for 55 min at a rotation speed of 200 rpm at 80 °C from an electrolyte consisting of 0.1 M Zn(NO_3_)_2_, 0.9 mM InCl_3_, and 0.4 M KCl; the pH bath was adjusted by NaOH to 3.5.

### 2.2. Instrumental Techniques

A Bio-Logic potentiostat/galvanostat Model VSP A (Seyssinet-Pariset, France) was used as the power source to obtain polarization curves by applying a constant step voltage (chronoamperometry experiments) on the electrochemical system. The final electrodeposit composition at the CIGS surface was determined using a Hitachi S4500 scanning electron microscope (SEM) equipped with a Noran energy dispersive spectrometer (EDS), (Santa Clara, CA, USA). The focused ion beam (FIB) for the atomic composition analysis in the cross section of the CIGS film was determined by the FEI Helios NanoLab 650 Dual-Beam System equipped with an EDS (Milpitas, CA, USA). KLA-Tencor P-6 Stylus Profilometer, (Milpitas, CA, USA) was used to determine the thickness of the CIGS film. X-ray diffraction (XRD) was used to analyze CIGS crystallography (Bruker Discover D8 X-ray diffractometer, Billerica, MA, USA) with a Cu K α (alpha) radiation (λ = 0.15406 nm) as the source, with a step of 0.01°. The equipment used in the PV measurements was the QEX10 quantum efficiency measurement system (PV Measurements, Boulder, CO, USA), and an Oriel Sol2A solar simulator, (Irvine, CA, USA). As a result, the characterized parameters were overall efficiency, quantum efficiency, open circuit voltage, short circuit current, dark current, band gap, and the fill factor.

### 2.3. CIGS Electrodeposition—Overriding Considerations

The large difference in the standard deposition potentials of the four co-deposited metals presents a major challenge to CIGS electrodeposition. The cathodic reactions and standard potentials for the four components are:

HSeO^+3^ + 4H^+^ + 4e^−^ + OH^−^ ⇌ H_2_SeO_3_ + 4H^+^ + 4e^−^ ⇌ Se + 3H_2_O (0.74 V vs. NHE) (1)

Cu^2+^ + 2e^−^ ⇌ Cu (0.34 V vs. NHE) (2)

In^3+^ + 3e^−^ ⇌ In (−I.34 V vs. NHE) (3)

Ga^3+^ + 3e^−^ ⇌ Ga (−G.53 V vs. NHE) (4)

The co-deposition must take place at a potential more negative than that of gallium (−0.53 V vs. NHE), leading to the deposition of all other species (Se, Cu, In), in which they reduce at a very cathodic voltage to their standard potential, and therefore, close to, or at their limiting current—a region where they are critically sensitive to transport and convection. The diffusion flux of ionic species j towards the electrode can be written as:

(5) $$N_{j}=\frac{D_{j}\left(C_{b,j}-C_{e,j}\right)}{\left(1-t_{j}\right)\delta_{j}}$$

where, *N*_j_, *D*_j_, and *t*_j_ are the ionic flux, the diffusivity, and the transport number of ionic species j, respectively. *C*_b,j_ and *C*_e,j_ are the bulk concentration and the concentration at the electrode of species j, respectively, and *δ*_j_ is its equivalent Nernst diffusion layer thickness [47]. The latter depends slightly on the ionic species, however, when the diffusion coefficients of the involved species do not differ greatly, we may assume that *δ* for each species is independent and depends mainly on the prevailing transport mode and conditions. In a well-supported electrolyte such as the CIGS system, the transport number can be zero, *t*_j_ ~ 0, and at the limiting current conditions the concentration at the electrode is zero, *C*_e_ = 0. As a result, Equation (5) yields the transport limited flux condition, *N*_j,L_:

(6) $$N_{j}=\frac{D_{j}C_{b,j}}{\delta_{j}}$$

Therefore, the corresponding limiting current density can be written as follows:

(7) $$i_{j,L}=\frac{n_{j}F\;D_{j}C_{b,j}}{\delta_{j}}$$

where *n*_j_ is the number of electrons transferred in the electrode reaction and F is Faraday’s constant.

Accordingly, we expect that the mass transport of selenium, copper, and indium will be dominated in the electrodeposition process; consequently, agitation and convective flow play an important role. It should also be stated that just about all commercial plating processes are carried out under some convective flow mode, typically pumping, air agitation, or translating electrodes. However, because of the challenge in quantifying and scaling those processes, and because the deposit texture produced under mass transport control is typically rough and powdery, the design of most practical processes is under kinetics control.

## 3. Results

Initially, deposition of the CIGS layer was carried out from the conventional electrolyte composition. However, the deposit was rough and powdery and did not adhere well to the electrode. This was noted particularly at the higher rotation rates (250 to 600 rpm) where the deposit from the conventional electrolyte was shed as powder off the electrode into the solution. This is further discussed, and samples are shown, subsequently. 

To improve the quality of the deposit, a dilute electrolyte for CIGS electrodeposition was introduced. This considerably more dilute solution (about ten-fold) yielded significantly improved deposits with the expected composition. The dilute electrolyte produced a smooth deposit with no evidence of powder formation on either the substrate or within the electrolyte, across a broad rotation range up to 600 rpm. In this lower concentration system, the desirable final composition was obtained in the potential interval −0.66 ≤ E ≤ −0.76 V vs. NHE providing uniform atomic composition across the sample. 

### 3.1. Polarization Studies in CIGS

Polarization studies were conducted on the RDE of stainless steel covered with a molybdenum layer obtained with a sputtering process previously described in Section 2.1. These were to perform an electrodeposition process for both electrolyte systems: the conventional concentrated system (~10^−3^ M), and the newly developed dilute system (~10^−4^ M). The quaternary (CIGS) and the binary (CuSe) systems were specifically studied.

**Figure 1:**
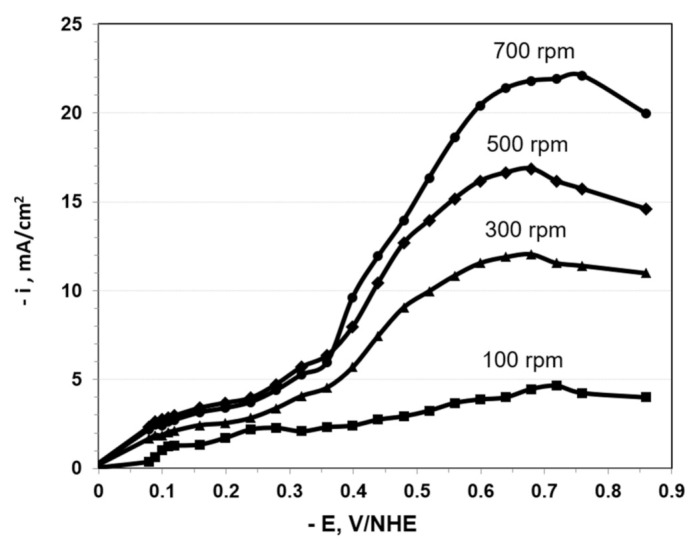
Polarization curves for CIGS electrodeposition on a disk electrode rotated at the indicated rates, from the conventional, higher concentration bath that consisted of 5 mM CuCl_2_·2H_2_O; 5.3 mM InCl_3_; 7.8 mM H_2_SeO_3_; 6.1 mM GaCl_3_; pHydrion (pH = 2); and 0.66 M LiCl as supporting electrolyte. The electrodeposition was carried out at ambient temperature = 20 °C.

The polarization curves obtained for the higher concentration system (Figure 1) demonstrate the difficulties in defining the limiting current, due to a large amount of hydrogen co-evolution associated with the process. We believe, based on visual observations, that the reduction in current past the peak at about −0.75 V vs. NHE is due to a copious amount of co-evolved hydrogen, accumulating as bubbles on the electrode and blocking a portion of its active area. As shown subsequently, the partial current densities were at the limiting currents for a number of the components such as Cu and Se, leading to rough deposits which exhibited pure adhesion and flaking. At the lower current densities and overpotentials, the current density increased rapidly due mainly to roughness; however, at higher potentials, the current density decreased due to bubble blockage.

**Figure 2:**
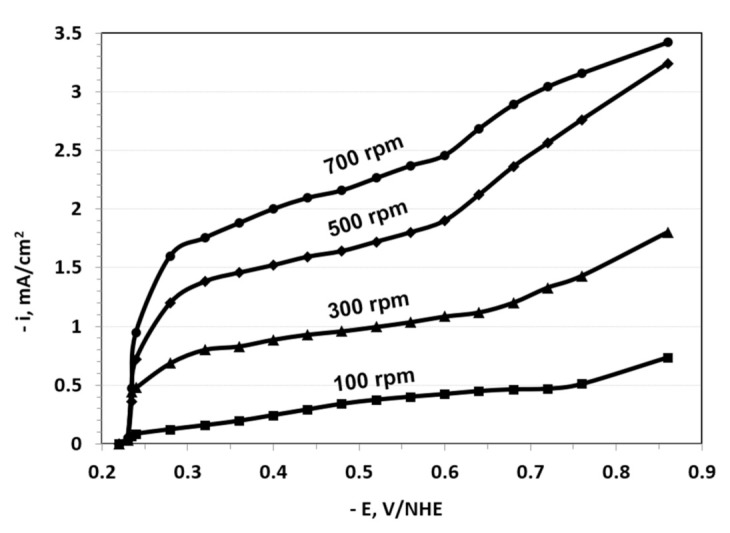
Polarization curves for CIGS electrodeposition on a disk electrode rotated at the indicated rates, from the low concentration bath consisting of 0.45 mM CuCl_2_·2H_2_O; 0.44 mM InCl_3_; 0.85 mM H_2_SeO_3_; 0.5 mM GaCl_3_; pHydrion (pH = 2); and 0.66 M LiCl as supporting electrolyte. The electrodeposition was done at ambient temperature = 20 °C.

By contrast, the polarization curves obtained for the lower concentration (by a factor of about 10) electrolyte exhibited better defined plateaus for each rotation rate, possibly because of less roughness in the formation of the film, since the deposit was smooth and adherent. As expected, the deposition current density from the lower concentration electrolyte was significantly lower than the current in the higher concentration system. Under those conditions, the process was below the limiting currents of indium and gallium. Additionally, a shift in the open circuit potential of about 300 mV vs. NHE (in the cathodic direction) was observed at the lower concentration system as compared to the higher concentration systems, most likely due to copper-selenium complexation (Figure 2).

### 3.2. Copper-Selenium Co-Deposition

**Figure 3:**
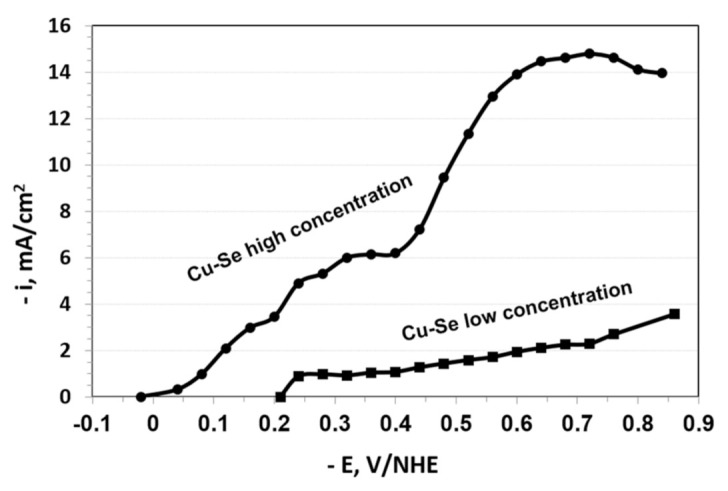
Polarization curves for copper and selenium co-deposition from the higher and lower concentration systems at 300 rpm. The electrolyte compositions were identical to those listed in Figure 1 (high concentration) and Figure 2 (low concentration) of the bath. Temperature = 20 °C.

Copper and selenium polarization curves were measured to investigate the cause for the shift of the open circuit potential (*E*_ocp_) between the higher and lower concentration systems by about 300 mV vs. NHE. The copper-selenium system showed (Figure 3) a shift of 0.23 V vs. NHE in the open circuit potential between the two systems. This shift is poorly known; thus, it may be due to the formation of secondary phases, in this case the phases could be In*_x_*Se or Cu*_y_*Se, among others [39]; the phases In*_x_*Se and Cu*_y_*Se could be formed, as well as the formation of CuInSe_2_. As a result, this spontaneous process generates complexity in the reaction mechanism implicated [38]. This shift may be due to another possibility in a different complexion of the pHydrion buffer to both selenium and copper ions, leading to different ion concentration at the electrode not being able to dismiss this shift on the open circuit potential.

### 3.3. Quantifying the Convective Flow Effects on CIGS

In order to determine mass transport effects on the CIGS electrodeposition, the polarization curves for both the conventional electrolyte and the new, low concentration electrolyte were measured on the RDE at three different rotation speeds, as shown in Figure 1 and Figure 2. To avoid a transient response, the polarization curves were generated by holding the potential at a given value until the current stabilized, and after recording this value, the potential was stepped up to the next measurement point. Figure 1 and Figure 2 display quite different current density scales. Both plots indicate that the current density increased with the rotation rate; however, while the conventional electrolyte exhibited a maximum current density at about −0.65 V vs. NHE, the lower concentration of the bath exhibited an increment in the current density across the entire scanned potential range.

In Table 1, the atomic composition of the surface of the film obtained after 40 min at −0.76 V vs. NHE at the three rotation rates (500, 300 and 100) rpm is shown for the lower and higher concentration baths described in Figure 1 and Figure 2. These electrodeposits were obtained at ambient temperature, 20 °C. The electrodeposit composition was analyzed using a Hitachi S4500 scanning electron microscope (SEM) equipped with a Noran energy dispersive spectrometer (EDS).

In the comparison of the deposited films of these two baths, it is possible to observe that the deposit composition was quite sensitive to the mass transport conditions when it was electrodeposited from the conventional electrolyte, with the copper and selenium concentrations increasing with the rotation rates while the indium and gallium content decreased. However, the films deposited from the new bath, low concentration of the precursors, were relatively insensitive to the rotation rate with the copper and selenium content in the deposit varying by less than 10% when the rotation rate was increased from 100 to 500 rpm.

Comparing experimental data displayed in Figure 1 to the calculated limiting currents of the four species in the higher concentration system, we found that both the selenium and the copper were electrodeposited at their approximate limiting currents (1.49 and 1.22 mA/cm^2^, respectively for 100 rpm, as given by Equation (3)) while indium and gallium were kinetically controlled. Therefore, it is proven that mass transport has an important role in CIGS electrodeposition due to metal atomic ratio variation at different rotation speeds. Since the copper and selenium are electrodeposited at their limiting currents, these elements will be more sensitive to rotation-induced mass transport than the kinetically controlled indium and gallium.

By contrast, the lower concentration system exhibited less dependence of the deposit composition on the rotation speed, as indicated in Figure 2. For example, the selenium content in the deposit increased by just about 2% when there was an increasing rotation speed from 300 to 500 rpm. At the low concentration, all metal species were mass transport controlled (Figure 2) and hence they were all affected similarly by the rotation speed; as a result, the composition of the film was not greatly affected by the rotation speed.

### 3.4. Hydrogen Evolution

**Figure 4:**
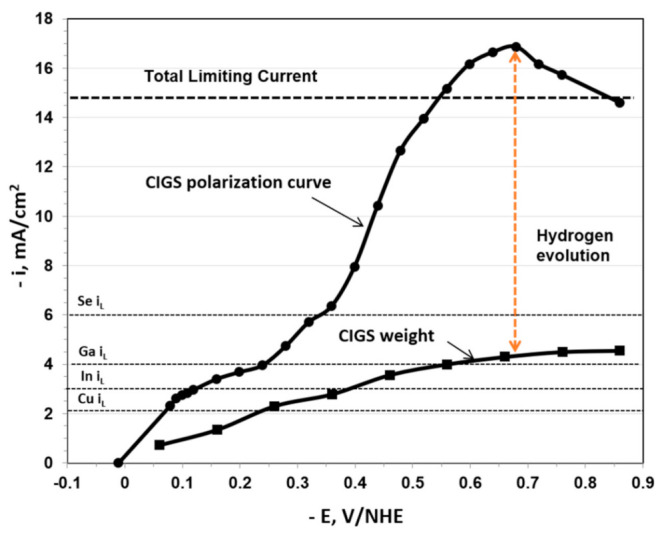
Polarization curves for CIGS deposition from the higher concentration system at 500 rpm. Subtracting the current density of the system by the current density obtained by the weight of the film through Faraday’s law provides the current density of hydrogen evolution. The indicated dashed lines are the expected limiting currents based on the non-complexed species concentration obtained with the Levich eq.

**Figure 5:**
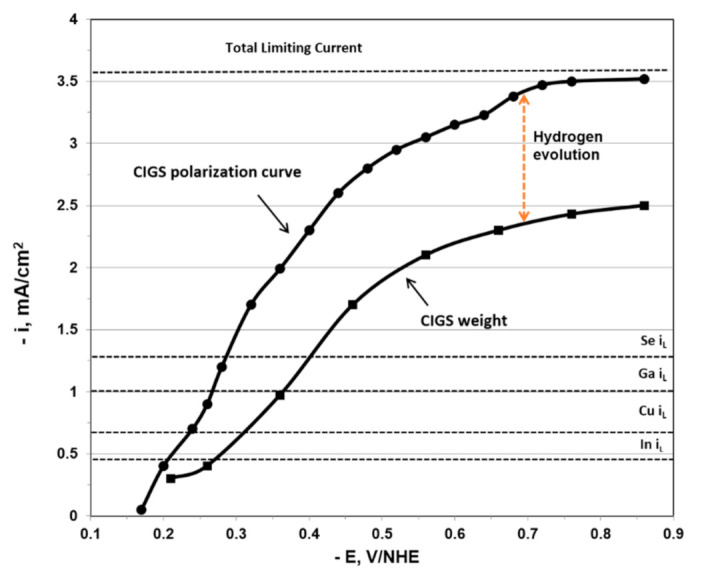
Polarization curves for CIGS deposition from the lower concentration system at 500 rpm. The difference between the overall measured current and the current based on partial currents determined from the deposit weight is due to hydrogen evolution. The electrolyte composition was identical to that listed. The indicated dashed lines are the expected limiting currents based on the non-complexed species concentration obtained with the Levich eq.

In order to characterize the magnitude of hydrogen evolution and gain insight into the controlling deposition regime of the individual components, partial current polarization curves were assembled by analyzing the sample composition (by EDS) and then determining the partial currents from the individual metal components weight using Faraday’s law. The partial currents polarization curves are shown in Figure 4 and Figure 5.

The partial currents data were combined to display the total CIGS deposition current density, based on the compositional analysis of the deposit, as displayed in Figure 4 and Figure 5. The difference between the measured total current density and the one based on the compositional deposit analysis is ascribed to hydrogen evolution. Also indicated in Figure 4 and Figure 5 are the theoretical limiting current densities *i*_L_ determined using the Levich equation shown below [45]:

(8) $$i_{L}=i_{\mathrm{Levich}}=0.62nFAD_{j}^{2/3}\omega^{1/2}\upsilon^{-1/6}C_{b,j}$$

Equation (8) is the expression of limiting current density on a RDE under steady state conditions. Here, *ω* is the angular rotation rate (rad/s), *υ* is the kinematic viscosity of the electrolyte (≈0.01 cm^2^/s), and A is the geometric axial area of the working electrode.

The polarization curves associated with the higher concentration system (Figure 4) displayed significantly higher observed current density than expected indicative of a very large amount of hydrogen evolution, accounting for about 75% of the total current. It should be noted that the maximum hydrogen evolution occurred at about the same potential where the maximum total current was observed. At this peak, the current efficiency was about 23%. The hydrogen evolution declined at higher overpotentials, possibly due to bubble blockage of the electrode, as previously discussed.

The corresponding polarization curves, depicting the hydrogen evolution in the lower concentration system (Figure 5) indicate significantly lower hydrogen evolution as compared to the higher concentration system, both on the absolute scale and as a fraction of the actual deposition current. The amount of evolved hydrogen corresponds in this system to only about 20% of the total current. The smooth deposit and the difference in electrochemical behavior between the two systems may be explained by the amount of hydrogen gas evolved on the surface. This means that the morphology of the surface deposit can be affected by the presence of hydrogen evolution and the concentration of the metal ions since all these species are competing to be reduced at the electrode surface.

A number of common baths that have been described earlier in the literature with composition ranges listed in Table 2 were also investigated. The deposit composition was controlled by adjusting the pH, the overpotential, and the species concentrations in the baths. The deposits were mostly in the desired range for CIGS composition. However, it was difficult to control the process while applying agitation, especially in the 300 to 600 rpm range. The deposit was rough and tended to peel off. By contrast, the new electrolyte introduced herein, where the concentrations of the major components were reduced by a factor of about 10 as compared to the conventional baths, produced high-quality films. The deposit was smooth and adherent across the entire tested rotation range of 0 to 700 rpm and its composition was uniform across the substrate and did not vary much with agitation.

Table 3 indicates the minimum and maximum concentration for each species to provide the final composition. Table 3 lists the deposit weight and its adhesion quality as a function of the disk rotation rate and the current density, for the two systems of the higher and the lower concentrations. Electrodeposition was carried out at a constant potential of −0.76 V vs. NHE at room temperature (20 °C) for 40 min. The metals composition in the electrolyte was adjusted to obtain the desirable CIGS final stoichiometry.

The stainless steel disk was weighed before and after the deposition in order to determine the deposit weight. As noted in Table 3, less weight was in fact measured at higher rotation rates (>300 rpm) for the conventional, high concentration system as compared to the low concentration system. This unexpected result is due to the non-adherent nature of the deposit obtained from the high concentration electrolyte, which peeled off the substrate at the higher rotation speeds.

### 3.5. Peel Test

**Figure 6:**
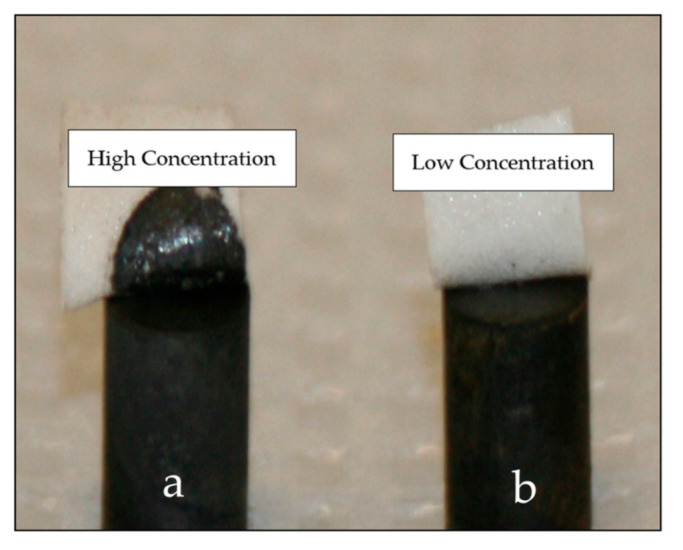
Peel test results, comparing adhesion of CIGS deposits. (**a**) electrodeposit obtained with the bath with high concentration. (**b**) electrodeposit obtained with the bath with low concentration. The substrate was a stainless steel cylinder, 0.32 cm in diameter, electrodeposited on its flat end. A deposit of 1.6 μm CIGS was electrodeposited at −0.76 V vs. NHE for 40 min at 500 rpm. The stainless steel substrate was cleaned in acetone and etched for 2 min in 10% with sulfuric acid prior to deposition.

Peel tests were applied to the deposits from both systems (Figure 6). The peel test consisted of applying scotch tape to the deposit (disk with a radius of 0.32 cm) and peeling it rapidly. The deposit was considered to be of a good quality if it remained intact. Deposit fragments observed on the peeled tape were indicative of poor adhesion. Six of the seven samples electrodeposited from the low concentration system passed the peel test successfully, while all seven samples from the high concentration solutions failed the test (Figure 6). The peeled tape on the cylinder (a) (high concentration) shows a significant cylindrical black patch of peeled deposit, while the tape on the cylinder (b) (low concentration) is clear.

### 3.6. Deposit Morphology

**Figure 7:**
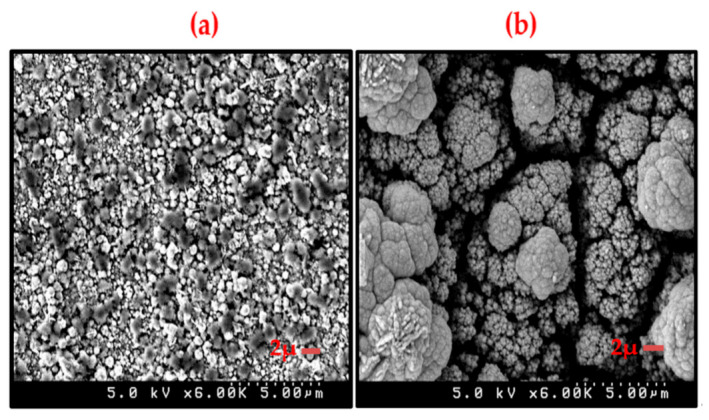
SEM micrographs of CIGS electrodeposited samples. (**a**): deposit from the lower concentration electrolyte. (**b**): deposit from the higher concentration electrolyte. The crystalline size in the deposit from the new, low concentration electrolyte was of the order of 1 μm and exhibited only moderate size distribution. By contrast, the deposit from the conventional, concentrated electrolyte exhibited a much larger crystalline size (10 μm) with a large size distribution and discontinuities. The electrodeposition conditions and substrate preparation were identical to those listed for Figure 6.

SEM inspection of the deposit reveals that the deposit from the lower concentration system had a quite small nuclei size, on the order of 1 μm. Additionally, the deposit provided continuous coverage of the surface and exhibited a fairly narrow (one to two microns) distribution of nuclei size. By contrast, the deposit obtained from the higher concentration system had a much larger nuclei size, on the order of 10 μm. The deposit has a larger variation of nuclei size (5 to 20 μm). The most troublesome characteristic was the larger gaps observed in the deposit, indicating a cracked and rough deposit (Figure 7).

### 3.7. Electrolyte Composition and Potential Range for Obtaining the Desired CIGS Composition

To establish the effect of the applied potential on the deposit composition, electrodeposition studies were carried out in the range between −0.66 and −0.76 V vs. NHE at 500 rpm and ambient temperature. All deposition experiments were carried out for 40 min. The metals ion concentration in the electrolyte was adjusted in the different experiments under the different potentials to obtain the desirable CIGS final stoichiometric atomic composition. The second through the fourth columns in Table 4 list the solution compositions from which the near-optimal deposit compositions were obtained, under the potential listed in the fifth column. The last four columns on the right describe the final alloy atomic compositions as determined by Hitachi S4500 scanning electron microscope (SEM) equipped with a Noran energy dispersive spectrometer (EDS). It was difficult to obtain the desirable gallium amount in the final composition under all the tested conditions at −0.6 V vs. NHE. Good indium gallium distribution and a smooth layer were detected in the range of −0.64 to −0.76 V vs. NHE. The final composition listed was obtained after annealing for one hour at 520 °C under argon atmosphere.

### 3.8. Uniformity on the CIGS Electrodeposition

#### 3.8.1. CIGS Composition across Sample

**Figure 8:**
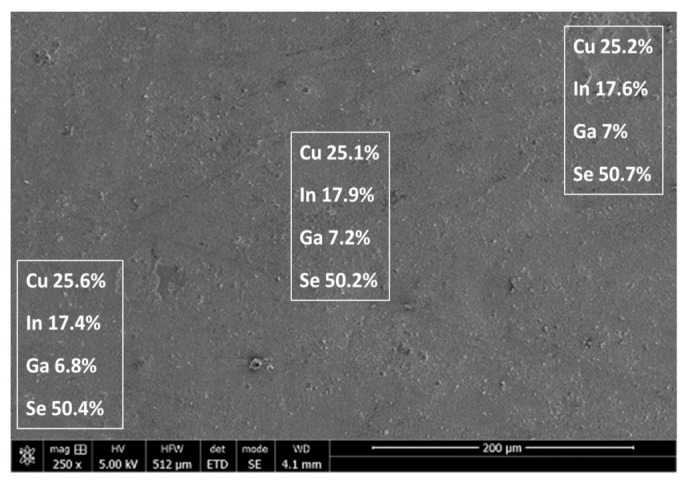
CIGS composition uniformity across a sample electrodeposited from the low concentration, new electrolyte. The listed compositions were measured by EDS at approximately the center of the corresponding rectangles. The sample was electrodeposited for 40 min at −0.76 V vs. NHE, on a disk rotating at 500 rpm, under ambient temperature (20 °C). The electrolyte composition was identical to that listed in Figure 2.

The analysis of the CIGS deposit (by EDS) indicated uniform composition across a 500 μm sample for all four species as illustrated in Figure 8. It is particularly significant to note that the gallium had a uniform composition, at the correct target value, prior to annealing. Typically, due to its high vapor pressure, it is difficult to obtain the optimum gallium composition, and adjusting it during annealing is likely to extend the eventual annealing time.

#### 3.8.2. CIGS Thin Film Composition by Depth Profiling

**Figure 9:**
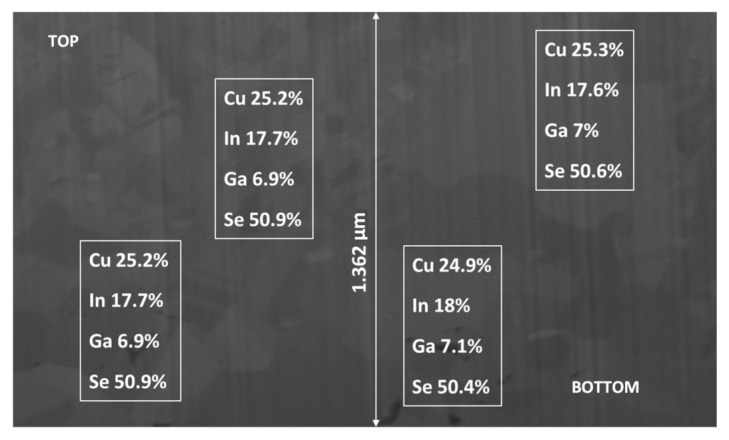
CIGS composition variation with depth as measured by FIB in a sample electrodeposited from the low concentration, new electrolyte. The listed compositions were measured at approximately the center of the corresponding rectangles. The sample was electrodeposited for 40 min at −0.76 V vs. NHE, on a disk rotating at 300 rpm, at ambient temperature (20 °C). The electrolyte composition was identical to that listed in Table 2.

The analysis of the CIGS samples was obtained using FIB. In detail, the atomic composition information in the cross-section was determined by the FEI Helios NanoLab 650 Dual-Beam System equipped with a EDS. As shown in Figure 9, uniform atomic composition throughout the sample depth was obtained for the four components. Gallium again showed uniform composition prior to annealing, thus potentially enabling the reduction of the annealing time. It should also be noted that both gallium and indium exhibited uniform distribution across the sample, thus enabling an increase of the gallium atomic content in the deposit to 9% to increase the energy band.

### 3.9. Effects of the Electrolyte Concentration on the Deposit Morphology and Adhesion

**Figure 10:**
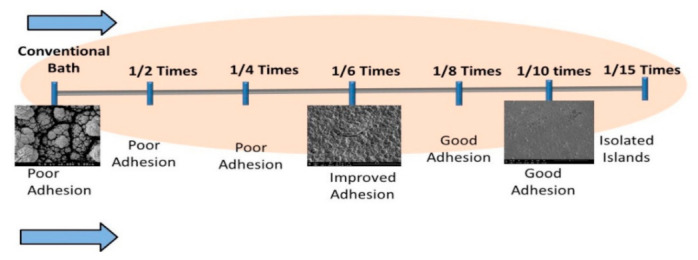
CIGS deposit quality as a function of the bath concentration. The indicated dilutions are with respect to the conventional bath composition. As noted, the best adhesion and deposit morphology was obtained at about 1/10 the concentration of the conventional electrolyte composition.

The deposit composition, morphology, and the electrochemical behavior (polarization curves) of the system were determined as a function of the concentration of the major components of the electrodeposited electrolyte in the ranges indicated in Figure 10 at fixed potential −0.76 V vs. NHE, rotation rates 600 rpm, and electrodeposition times 40 min. Deposits obtained from 1/2 and 1/4 of the conventional bath concentration (i.e., bath dilutions by factors of 2 and 4) were compared to deposits obtained from the conventional bath. It was observed that for these dilutions (1/2 and 1/4), the deposit was still rough; the electrochemical behavior (total current plateau, Figure 1 and Figure 3) of this system was still similar to that obtained from the conventional composition. At 1/8 of the conventional bath (eight-fold dilution), smooth deposit was obtained. Moreover, the shape of the polarization curve of this system was still similar to that of the new bath (total current plateau, Figure 2 and Figure 5).

At 1/15 of the conventional bath concentration (15-fold dilution), dark spots were noted on the deposit, indicating voids. Accordingly, it was determined that the best concentration range for CIGS electrodeposition is between 1/8 to the 1/10 of the conventional bath concentration.

### 3.10. Effects of the Electrolyte

Thermal annealing is typically applied after deposition to improve the deposited layer crystal structure, to decrease the recombination of defects, and to obtain a uniform atomic ratio [39,40,41,42,43,44,45,46,47,48,49,50]. The post treatment parameters depend on the thin film composition, its thickness, the partial vapor pressure, and on the binary stacked layers [51].

The thermal treatment step was carried out in a tube furnace at 520 °C under argon atmosphere for 30 min at a gas flow rate of 8 cm^3^/s. The temperature was selected to provide the optimal crystallinity for the CIGS absorber layer [1,37]. Low melting point indium selenides (In_x_Se_y_) are partially lost during the annealing process as indicated by the data in Table 5.

### 3.11. Crystallography of CIGS Thin Film

The deposited CIGS thin film was annealed at 500 °C for 30 min under argon atmosphere, and then subjected to XRD analysis to determine the crystallography of the absorber layer. The tested sample was 1.52 µm thick (KLA-Tencor P-6 Stylus Profilometer) (Milpitas, California, USA), and had a composition of 24.9% Cu, 17.3% In, 7.7% Ga, and 50.1% Se. The sample was electrodeposited at −0.76 V vs. NHE for 40 min, under ambient temperature (20 °C) on a stainless steel substrate disk rotated at 500 rpm. The electrolyte composition was 4.4 mM CuCl_2_·2H_2_O; 4.85 mM InCl_3_; 8.1 mM H_2_SeO_3_; 6.7 mM GaCl_3_; pHydrion (pH = 3); and 0.66 M LiCl as supporting electrolyte.

**Figure 11:**
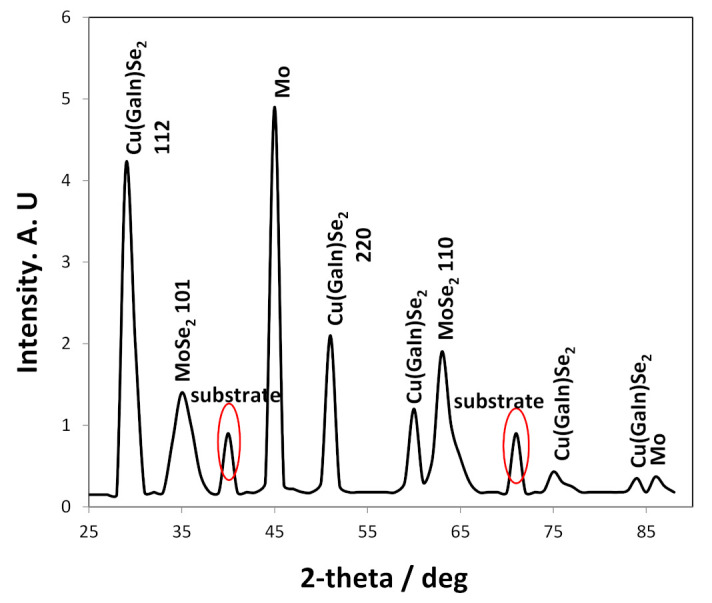
Diffraction peaks of the CIGS layer deposited from the low concentration electrolyte, after annealing for 30 min at 500 °C. The tabulated insets indicate the deposit composition obtained after 40 min at −0.76 V vs. NHE at 500 rpm. The electrolyte consisted of 0.45 mM CuCl_2_∙2H_2_O; 0.44 mM InCl_3_; 0.85 mM H_2_SeO_3_; 0.5 mM GaCl_3_; pHydrion (pH = 2); and 0.66 M LiCl as supporting electrolyte. Temperature = 20 °C.

Two main diffraction peaks associated with <112> and <220> planes that correspond to the CIGS crystal structure are observed in Figure 11. The XRD analysis showed that the deposited absorber thin film had a Cu(GaIn)Se_2_ structure, with no other peaks detected. Likewise, the most intense diffraction peak <112> was located at 26.81°, which is indicative of the desirable CIGS crystallography due to the crystal phase corresponding to chalcopyrite material (JCPDS card 35–1102). The thickness of the CIGS layer was about 1.4 µm, which should be sufficient to absorb 97% of the incident light.

### 3.12. Mechanistic Discussion of Surface Coverage and Nucleation Density

**Figure 12:**
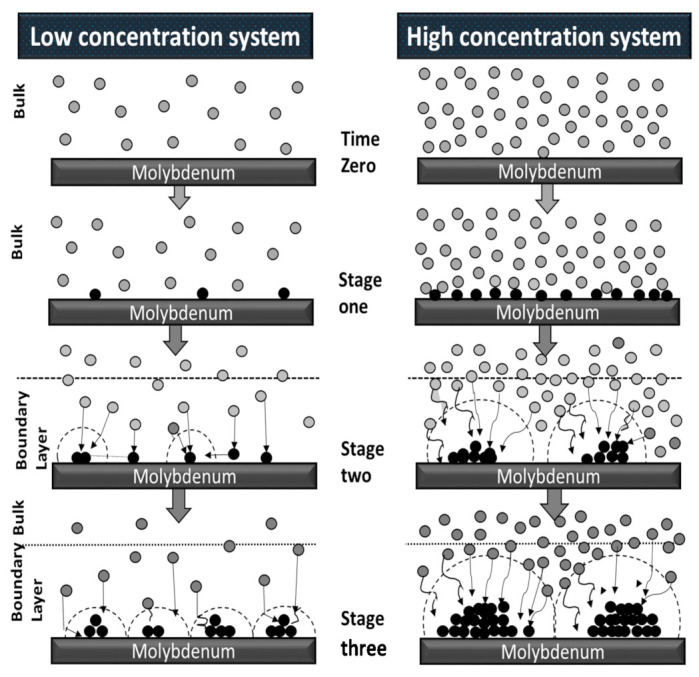
Schematic illustrating the three steps of CIGS electronucleation at the molecular level following the Scharifker/Hills model. Stage one shows more ions near the substrate surface at the high concentration system as compared to the lower concertation system. Stage two indicates that adatoms start to accumulate at the higher concentration system while the adatoms still travel longer in the low concentration system. In stage three, the higher concentration system displays larger nuclei as compared to the lower concentration system.

A model proposed by Scharifker and Hills [52], providing a relationship between the nucleation density and the electrolyte concentration, was adapted to analyze the process and results of the present research. Figure 12 is a schematic representation of probable steps in the deposit nucleation process. The two panels provide a suggested comparison for the effect of the electrolyte concentration on the deposit morphology.

At the initial stage of the electrodeposition process, the number of adatoms deposited on the substrate surface is a function of the electrolyte bulk concentration [52], which is larger for the higher concentration electrolyte. Consequently, the distance between metal adatoms is larger in the low concentration system as compared to the higher concentration system. The adatoms migrate towards one another to reach a minimal energy state. In order to form nuclei in the lower concentration system, adatoms need to travel a longer distance as compared to the high concentration system. Therefore, in the low concentration system, the adatoms may reach the minimal energy state (stop moving) before meeting the closest adatoms, resulting in a large number of small nuclei [45]. In contrast, in the higher concentration system, since the density of electrodeposited adatoms on the surface is high, the closest adatoms are likely to group together to create larger nuclei, resulting in a lower nuclei population density [52].

The Scharifker/Hills model uses the peak of transient current (*I*_max_) and corresponding peak time (*t*_max_) at different metals concentrations to determine the nuclei population density for the system [52]. Accordingly, the nuclei population density is given by:

(9) $$N_{0}=0.065{\left(\frac{1}{8\pi C_{0}V_{m}}\right)}^{1/2}{\left(\frac{nFC_{0}}{i_{\max}\cdot t_{\max}}\right)}^{2}$$

Here, *C*_0_ is the concentration of species in the bulk; *V*_m_ is the molar volume; *t*_max_ is the peak time; and *i*_max_ is the peak current density (A/cm^2^).

When substituting the corresponding numerical values into the Scharifker/Hills model, we find that the expected nucleation density for the conventional bath, higher concentration system was 2 × 10^6^ nuclei/cm^2^, while the lower concentration system (1/10 dilution) was expected to yield a far lower 1.5 × 10^8^ nuclei/cm^2^. This was in close agreement to measurements of deposits from the low concentration electrolyte, which indicated nuclei density of about 3 × 10^8^ nuclei/cm^2^. Clearly, the higher nuclei density is expected to provide better surface coverage and improved adhesion. A description to explain why it is better to perform electrodeposition experiments at lower concentrations to inhibit roughness and enhance adhesion on the film can be explained in Figure 12. Here, it is possible to notice that in a higher concentration system, the presence of large clusters in scattered areas of the surface is favored, thereby proving heterogeneity in the composition of the alloy since not all precursors follow the same dynamic; that is, some of the precursors are governed by mass transfer while others are controlled by a kinetics reaction to the potential applied to form the CIGS quaternary alloy.

### 3.13. Optical Characterization Test

**Figure 13:**
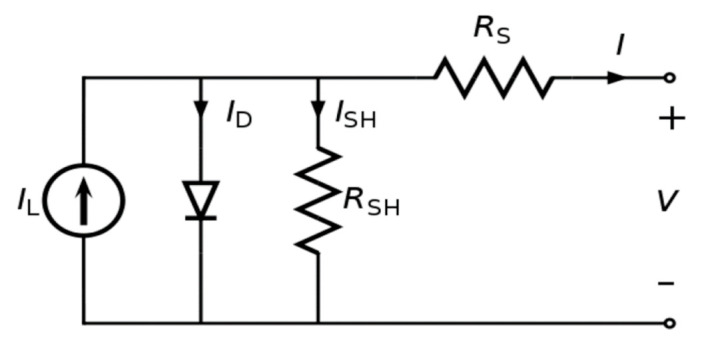
A typical equivalent circuit of a PV cell.

This thin film semiconductor of CIGS study was done in an electrodeposition process of a single bath (one step) without the need to add a gas phase from an additional precursor, which is not reported in the literature. It is therefore relevant to evaluate if the alloy has a photovoltaic response. Thus, the characterizations tests were done on the CIGS device after its completion process. The solar cell equivalent is shown in Figure 13. The equivalent circuit is helpful for characterizing the electrical response of the device and identifying the important factors that may affect its behavior. The Illumination current (*I*_L_) is possibly the most important measured parameter in the solar cell and it is also called the photogenerated current. Another measured current is the dark current (*I*_D_), which is the reverse saturation current, and it is measured under dark conditions. Dark current represents a standard diode curve of the photovoltaic equivalent circle. The difference between the illuminating current and the dark current is the solar cell generated current [53].

The shunt resistance (*R*_SH_) is due to electron-hole recombination; in the shunt, resistance passes the shunt current (*I*_SH_); the series resistance (*R*_S_) is due to conductivity and connection wire imperfection. Consequently, output current (I) of the photovoltaic cell’s can be described as follow [54]:

(10) $$I=I_{L}-I_{D}\left\{exp\left[\frac{q\left(V+I\cdot R_{S}\right)}{Q_{D}kT}\right]-1\right\}-\frac{V+I\cdot R_{S}}{R_{SH}}$$

Hence, *Q*_D_ is the diode ideality factor, *q* is the elementary charge of an electron, *V* is the voltage across terminals, *T* is the absolute temperature of the PV cell, and *k* is the Boltzmann’s constant.

Furthermore, the connection device was designed in the lab in order to have a complete electric circuit to characterize the solar device. The characterized parameters were overall efficiency, quantum efficiency, open circuit voltage, short circuit current, dark current, band gap, and the fill factor. As a result, the equipment used was a PV Measurements QEX10 quantum efficiency (PV Measurements, Boulder, CO, USA) measurement system and an Oriel Sol2A solar simulator (Irvine, CA, USA). The solar simulator provides light and radiation similar to that provided from the Sun. The solar simulator tests the solar device efficiency and determines additional outcome parameters, including fill factor (*FF*), open circuit voltage (*V*_OC_), and the short circuit current (*I*_SC_). Fill factor is a parameter used to determine the maximum operating power point. It is given by:

(11) $$FF=\frac{P_{m}}{V_{OC}\cdot I_{SC}}=\frac{\eta \cdot A_{C}\cdot E}{V_{OC}\cdot I_{SC}}$$

where (*η*) is the solar cell’s energy conversion efficiency, (*E*) is the input power, and (*P*_m_) is the maximum power.

**Figure 14:**
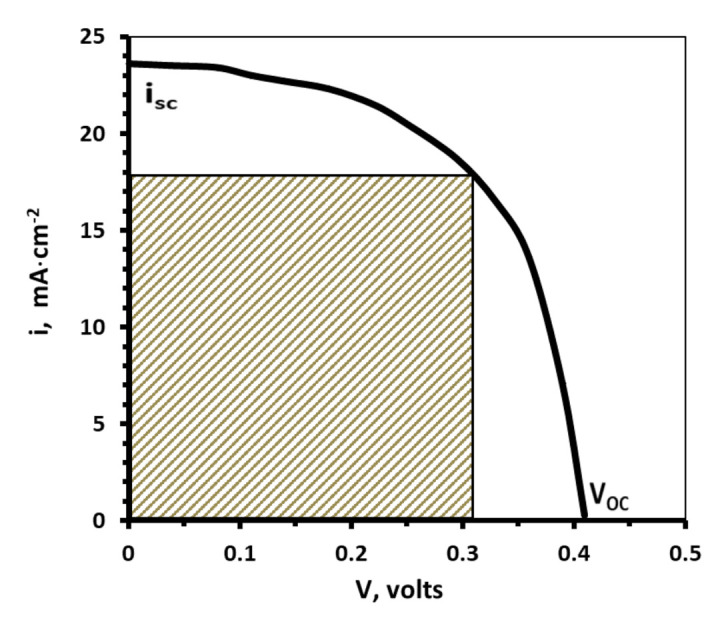
**“**Current density-voltage” (i vs. V) characteristics of SS/Mo/Ni/CIGS/CdS/i-ZnO/Al:ZnO structure.

In Figure 14, it is shown the “current density-voltage” (i vs. V) plot obtained with the set up described in Figure 13 and for the SS/Ni/Mo/CIGS/CdS/i-ZnO/Al:ZnO device; this device was prepared with the electrodeposition of CIGS of the lower concentration electrolyte.

The open short circuit current and open-circuit voltage were about 23.5 mA/cm^2^ and 0.41 V, respectively. The fill factor of the CIGS device was about 49 %.

**Figure 15:**
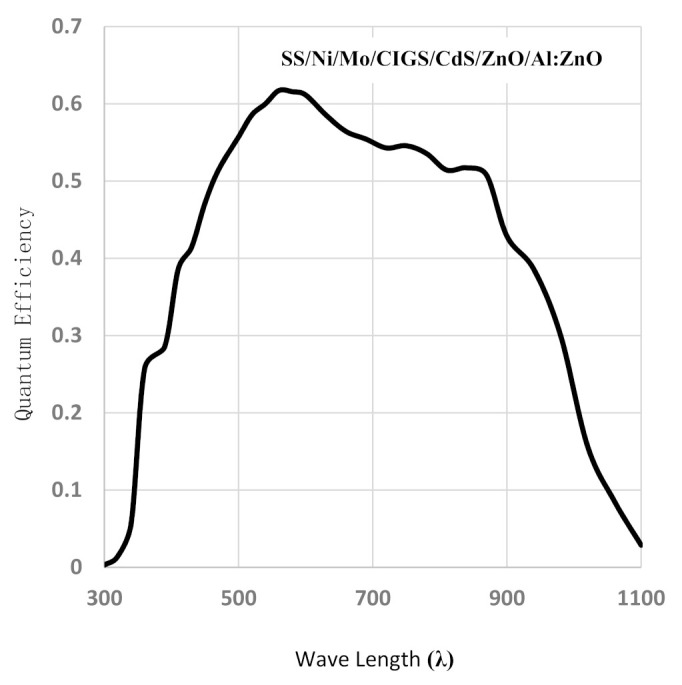
Quantum efficiency characteristics of the SS/Ni/Mo/CIGS/CdS/i-ZnO/Al:ZnO structure.

The external quantum efficiency is the ratio of exited electrons to incident photons. The current produced per incident photon was measured as a function of the corresponding wavelength. The quantum efficiency for CIGS solar device under illumination was done at AM1.5, (1000 W/m^2^), and it is presented in Figure 15. The quantum efficiency measurements collected is about 6% efficient as it is shown in Figure 15.

This characterization test proves that the CIGS absorbs a higher number of photons in the visible light spectrum than in the longer wavelength spectrum. The quantum efficiency was high because the CIGS thin film obtained from the low electrolyte concentration had good quality. The band gap calculations match the experimental one, which is about 1.29 eV. The quantum efficiency was about 0.62 at the optimal band gap as it is shown in Figure 15. Finally, in this study there was not an optical characterization of the CIGS thin film obtained from the higher concentration bath because this layer did not show a smooth morphology or adhesion with the back contact barrier.

## 4. Conclusions

A single step electroplating process for the deposition of high-quality quaternary CIGS absorber layer from a new, low concentration electrolyte composition has been demonstrated. The deposition process and the deposit composition as well as its properties have been characterized. The improved low concentration electrolyte, consisting of deposited ions in the 10^−4^ M range (corresponding to a dilution factor of about ten-fold in comparison to previously reported systems), provided an adherent and smooth deposit. Smaller nuclei sizes (~1 μm) at a density of about 3 × 10^8^ nuclei/cm^2^ with a uniform atomic composition at the optimal CIGS composition were observed across the samples, as well as in the deposit bulk. 

The flow effects on the deposit composition and its morphology were characterized for both the conventional and the new dilute electrolyte. It was determined that the transport conditions have a major effect on the deposit properties in both systems. The effect of the electrolyte composition and the applied voltage on the deposit composition has been characterized. The process parameters for the deposition of the CIGS absorber layer with near optimal composition have been identified. It was determined that unlike previously reported systems, there is no need for metal addition from the gas phase during the annealing process, and the annealing time under argon atmosphere could be significantly reduced (to about 30 min as compared to two hours in the conventional process).

Moreover, a weight analysis was done to describe the main role of the co-evolution of hydrogen gas in the electrodeposition process on the CIGS alloy. As a result, the presence of the hydrogen evolution in the quality and morphology of the electrodeposit film in which the electrochemical behavior changes is strongly associated with the presence of different concentrations of the precursors on the bath. Moreover, the optical characterization yielded results of an open short circuit current and open-circuit voltage about 23.5 mA/cm^2^ and 0.41 V, respectively. Likewise, the fill factor of the CIGS device was about 49%. In the quantum efficiency experiments, the device showed a value of 0.62 at the optical band gap.

Finally, this study and novel electrolyte system showed interesting results when obtaining a thin film electrodeposited with photovoltaic response in a single bath. Therefore, this study successfully provides the basis for designing an improved CIGS electrodeposition system on large areas required in commercial scales because the process has been conveniently governed by mass transfer.

## Figures and Tables

**Table 1:** Electrodeposit composition obtained after 40 min at −0.76 V vs. NHE at the three rotation rates. The higher concentration bath consisted of 5 mM CuCl_2_·2H_2_O; 5.3 mM InCl_3_; 7.8 mM H_2_SeO_3_; 6.1 mM GaCl_3_; pHydrion (pH = 2); and 0.66 M LiCl as supporting electrolyte. The lower concentration bath consisted of 0.45 mM CuCl_2_·2H_2_O; 0.44 mM InCl_3_; 0.85 mM H_2_SeO_3_; 0.5 mM GaCl_3_; pHydrion (pH = 2); and 0.66 M LiCl as supporting electrolyte. The electrodeposition process in both baths were carried out at ambient temperature, 20 °C.

Rotation Rate (rpm)		High Concentration Bath				Low Concentration Bath			
		Cu	In	Ga	Se	Cu	In	Ga	Se
500	%	30.1	8	4	57.6	24.3	12	6.8	56.9
	W (mg)	2.6	0.73	0.37	5	0.42	0.24	0.13	0.85
300	%	26.9	13	6.5	52	23.6	13.1	7.4	55.8
	W (mg)	1.6	0.7	0.36	2.9	0.36	0.22	0.11	0.86
100	%	21.2	20	11	44.1	21.8	15.1	9.5	53.1
	W (mg)	0.8	0.74	0.4	1.65	0.23	0.18	0.07	0.36

**Table 2:** Different CIGS electrolyte baths compositions reported in literature and the new chemistry introduced in this study.

Bath	CuCl_2_ (mM)	H_2_SeO_3_ (mM)	InCl_3_ (mM)	GaCl_3_ (mM)	Supporting	Potential	pH	ω
					Electrolyte	(V vs. SCE)		(rpm)
Literature, [14,15,16,17,18,19,20,21,22]	4–6	6–9	3–6	4–8	LiCl, Na_3_C_6_H_5_O_7_, pHydrion 2	−0.9 to −1	2–3	None
This work	0.4–0.6	0.7–0.9	0.3–0.5	0.3–0.9	pHydrion 2	−0.8 to −1.1	1–2	0–600

**Table 3:** Electrodeposited CIGS films at −0.76 V vs. NHE at the listed rotation speeds. (**a**) Left panels: high concentration system; (**b**) right panels: new, low concentration system. Adhesion was tested by a tape peel test. All tests were carried at ambient temperature (20 °C).

	(a) Conventional Composition (~10^−3^ M)			(b) New Composition, Dilute Electrolyte (~10^−4^ M)		
Rotation Rate (rpm)	i (mA/cm^2^)	Weight (mg)	Adhesion	i (mA/cm^2^)	Weight (mg)	Adhesion
0	1.42	0.7	Good	0.76	0.4	Good
100	3.98	1.1	Good	1.42	0.7	Good
200	6.8	1.2	Good	1.81	0.8	Good
300	9.11	1.1	Poor	2.02	1	Good
400	13.3	0.61	Poor	2.76	1.3	Good
500	17.27	0.32	Poor	2.91	1.5	Good

**Table 4:** CIGS composition after annealing as a function of deposition potential and bath composition. All deposition experiments were carried out at ambient temperature (20 °C) and a rotation rate of 500 rpm for 40 min.

	Potential	Electrolyte Concentration (mM)				CIGS Composition %			
Bath	V/NHE	CuCl_2_	GaCl_3_	InCl_3_	H_2_SeO_3_	Cu	In	Ga	Se
Bath 1	−0.76	0.5	0.37	0.32	0.845	24.5	17.4	7.6	50.3
Bath 2	−0.71	0.45	0.52	0.38	0.78	24.7	17.6	7	50.8
Bath 3	−0.68	0.45	0.69	0.47	0.77	25	17.8	7.1	50.1
Bath 4	−0.66	0.43	0.82	0.54	0.76	25.3	17.8	6.4	50.3
Bath 5	−0.64	0.42	0.9	0.61	0.76	25.4	17.7	6.3	50.2

**Table 5:** CIGS composition before and after annealing at 520 °C under argon atmosphere for 30 min at a gas flow rate of 8 cm^3^/s. Film obtained with the lower concentration bath described in Figure 1.

Metal	Before Annealing	After Annealing
Copper	22.9%	25%
Indium	16.8%	17%
Gallium	6%	7.5%
Selenium	54.3%	50.5%

## Data Availability

Data are contained within the article.
